# Identification of Anthocyanins-Related Glutathione S-Transferase (GST) Genes in the Genome of Cultivated Strawberry (*Fragaria* × *ananassa*)

**DOI:** 10.3390/ijms21228708

**Published:** 2020-11-18

**Authors:** Yuanxiu Lin, Lianxi Zhang, JiaHao Zhang, Yunting Zhang, Yan Wang, Qing Chen, Ya Luo, Yong Zhang, Mengyao Li, Xiaorong Wang, Haoru Tang

**Affiliations:** 1 College of Horticulture, Sichuan Agricultural University, Chengdu 611130, China; linyx@sicau.edu.cn (Y.L.); zlx981027@163.com (L.Z.); zhang2507374879@163.com (J.Z.); asyunting@gmail.com (Y.Z.); wangyanwxy@163.com (Y.W.); supnovel@gmail.com (Q.C.); luoya945@163.com (Y.L.); zhyong@sicau.edu.cn (Y.Z.); limy@sicau.edu.cn (M.L.); wangxr@sicau.edu.cn (X.W.); 2 Institute of Pomology and Olericulture, Sichuan Agricultural University, Chengdu 611130, China

**Keywords:** anthocyanins, cultivated strawberry, glutathione S-transferase, expression profiles

## Abstract

Anthocyanins are responsible for the red color of strawberry, they are a subclass of flavonoids synthesized in cytosol and transferred to vacuole to form the visible color. Previous studies in model and ornamental plants indicated members of the glutathione S-transferase (*GST*) gene family were involved in vacuolar accumulation of anthocyanins. In the present study, a total of 130 *FaGST* genes were identified in the genome of cultivated strawberry (*Fragaria* × *ananassa*), which were unevenly distributed across the 28 chromosomes from the four subgenomes. Evolutionary analysis revealed the expansion of *FaGST* family was under stable selection and mainly drove by WGD/segmental duplication event. Classification and phylogenetic analysis indicated that all the *FaGST* genes were clarified into seven subclasses, among which *FaGST1*, *FaGST37*, and *FaGST97* belonging to Phi class were closely related to *FvRAP*, an anthocyanin-related *GST* of wildwood strawberry, and this clade was clustered with other known anthocyanin-related *GSTs*. RNAseq-based expression analysis at different developmental stages of strawberry revealed that the expression of *FaGST1*, *FaGST37*, *FaGST39*, *FaGST73*, and *FaGST97* was gradually increased during the fruit ripening, consistent with the anthocyanins accumulation. These expression patterns of those five *FaGST* genes were also significantly correlated with those of other anthocyanin biosynthetic genes such as *FaCHI*, *FaCHS*, and *FaANS*, as well as anthocyanin regulatory gene *FaMYB10*. These results indicated *FaGST1*, *FaGST37*, *FaGST39*, *FaGST73*, and *FaGST97* may function in vacuolar anthocyanin accumulation in cultivated strawberry.

## 1. Introduction

Strawberry (*Fragaria* × *ananassa*) has been cultivated worldwide as one of the major fruit crops, it is highly regarded in the fresh fruit market mainly because of its attractive color, good flavor, and enrichment of nutrients. Particularly, the enriched anthocyanins, a class of water-soluble pigments, are not only responsible for the strawberry fruit coloration [1], but also one of the most important antioxidants and thereby contributing to the healthful attributes in strawberries [2,3].

Anthocyanins belonging to the flavonoid class of compounds are synthesized along with flavanols from phenylalanine and malonyl-CoA [4], proceeding in the cytosol. It has been suggested that any step of the enzymatic biosynthetic catabolites being upregulated or blocked could result in different colored plants. For instance, overexpression of *MaF3′H* from apple in Arabidopsis and tobacco resulted in red seedlings and flowers, respectively [5], while silencing of *FaDFR* [6] or *FaF3H* [7] in strawberry has resulted in complete loss of red color in fruits. Besides these enzymatic biosynthesis reactions, the transcriptional regulators of anthocyanin accumulation also have been well studied, especially the ‘MBW’ complex consisting of MYB, basic helix-loop-helix (bHLH), and WD40-repeat proteins (WD) [8,9]. A large number of studies have been conducted to identify the members of ‘MBW’ complex and reveal the molecular mechanism of anthocyanins accumulation regulation in plants [10,11,12]. However, to exhibit the visual color, anthocyanins must be transferred and stored into the vacuole with an acidic environment after synthesis [13]. Several types of proteins have been demonstrated as anthocyanins transporters in plants, such as multidrug resistance-associated protein (MRP) [14], multidrug and toxic extrusion (MATE) family [15,16,17], ATP-binding cassette (ABC) proteins [18], and probably the allergen *Fra a* 1 [19]. In recent years, evidence has shown that glutathione S-reductases (GSTs) are involved in anthocyanins transportation.

GSTs (EC 2.5.1.18) are encoded by a large gene family in plants, it comprises multifunction including detoxification of xenobiotics and stress responses [20,21]. According to the sequence identities, immunological characterizations, kinetic properties, and genomic structural organization, plant GSTs are classified into 10 subclasses including Phi, Tau, Lambda, Dehydroascorbate Reductase (DHAR), Tetrachloro hydroquinone (TCHQD), Elongation Factore-1 Gamma (EF1G), Zeta, Theta, Hemerythrin, and Iota [22]. Among which, the Phi class is plant-specific and recently suggested to be involved in anthocyanin transportation [23]. Subsequently, by using gene knockout and mutagenesis methods, GSTs have been demonstrated as the probable most important transporters of anthocyanins. For example, knockout of a single *GST* (*Bronze*-2, *Bz2*) gene in maize not only caused a large decrease of anthocyanins, but also loss the visible color of maize due to the loss of anthocyanins transport [14]. Similarly, several studies have suggested that the loss of *GSTs* function could result in the loss of visible pigment accumulation in plants, such as *PhAN9* in petunia [24], *VvGST4* in grape [25], *CkmGST4* in cyclamen [26], *LcGST4* in litchi [27], *MdGST* in apple [28], *Riant* in peach [29], and *AtTT19* in Arabidopsis [23]. The *AtTT19* has been further shown to act as a carrier protein for transportation of anthocyanins from cytosol into vacuole [23]. More recently, Luo et al. [30] have suggested that *reduced anthocyanins in petioles* (*rap*) encoded a GST transporter for anthocyanin that is essential for the woodland strawberry foliage and fruit coloration. However, the genome-wide identification of *GSTs* is limited in strawberry, a useful plant material for anthocyanins research. In the present study, the entire *GSTs* in the cultivated strawberry were identified genome-wide and analyzed. We further identified the potential anthocyanins-related *GSTs* based on our phylogenetic analysis, expression analysis, and correlation analysis. Our results here will provide candidate *GST* transporter genes for anthocyanins and thus will help the research for improving and manipulating anthocyanins accumulation in strawberry and other *Roceace* species in the future.

## 2. Results

### 2.1. Identification, Characteristics, and Evolutionary Analysis of the Entire 130 FaGST Genes in Cultivated Strawberry

Based on the genome searching and domain confirmation, totally, 130 *GST* genes containing GST domains were identified as *FaGST* genes from the whole genome of cultivated strawberry. All the 130 *FaGST* genes were renamed based on the order of their chromosome location (supplementary Figure S1), the gene names and gene IDs are listed in supplementary Table S1. As the results showed (Figure S1), 130 *FaGST* genes were unevenly distributed across the 28 chromosomes from the four subgenomes of cultivated strawberry, ranging from 1 to 11 genes per chromosome. A maximum 11 *GST* genes were located on chromosome 7 from the second subgenome (Fvb7-2), followed by 10 genes on chromosome 7 from the first subgenome (Fvb7-1). While only one gene was located on chromosomes Fvb4-1, Fvb1-2, Fvb6-3, and Fvb6-4, no *FaGST* was located on chromosome Fvb1-3. The characteristics of the 130 FaGST proteins are also shown in Table S1. The lengths of the protein sequences varied from 111 to 1137 aa, and most of them were from 200 to 400 aa. The protein molecular weights (MW) were from 12.73 to 129.14 KDa, and the isoelectric points (pI) were concentrated from 4.33 to 9.99. There was only one *FaGST* gene (*FaGST90*) which had a signal peptide, which is essential for secreted proteins. Most of the *FaGST* genes were predicted to be located in the cytoplasm, also some *FaGST* genes were involved in mitochondria and nucleus.

To elucidate the origin of *FaGST* genes in cultivated strawberry, the origin of duplication events was detected by MCScanX package. The results (Table S1) showed that four types of duplication events drove the expansion of *FaGST* genes including WGD/segmental, dispersed, tandem, and proximal. There were four genes (*FaGST6*, *FaGST7*, *FaGST36*, and *FaGST58*) duplicated from proximal events, eight genes named *FaGST73*, *FaGST68*, *FaGST80*, *FaGST97*, *FaGST94*, *FaGST104*, *FaGST101*, and *FaGST103* were involved in dispersed duplication. There were five couples of tandem duplication genes including *FaGST20*/*FaGST21*, *FaGST48*/*FaGST49*, *FaGST79*/*FaGST80*, *FaGST89/FaGST90*, and *FaGST82/FaGST83*.

The number of nonsynonymous substitutions per nonsynonymous sites (Ka), the number of synonymous substitutions per synonymous sites (Ks), and the Ka/Ks ratio for *FaGST* genes were calculated by Ka/Ks calculator (Table 1). The results showed, Ka ranged between 0.0017 to 0.919, Ks range was 0.01–0.989 for the identified paralogs (Table 1). Using the formula Ka/Ks, it was predicted that most *FaGST* paralogs might be under stabilizing selection, since the Ka/Ks was below 1. Except for the *FaGST36/FaGST38*, which had a Ka/Ks value above 1, indicating they were under positive selection.

### 2.2. Gene Ontology and Classification of FaGST Genes

**Figure 1:**
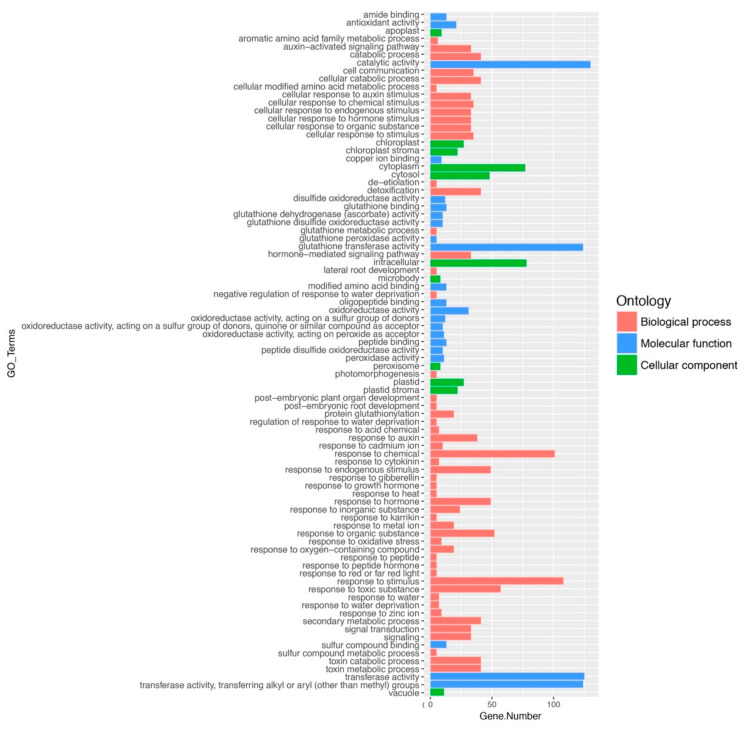
GO ontology of 130 *FaGST* genes. The vertical axis indicates the GO terms, the horizontal axis indicates the gene numbers in each term. The different colors of the bars indicate different GO categories.

To explore the functions of *FaGST* genes, gene ontology was carried out using Fa genome as background. As results (Figure 1, Table S1), 130 *FaGST* genes were significantly enriched in 22 GO categories of molecular function (MF), 11 GO categories of cellular component (CC), and 53 GO categories of biological process (BP). Among the MF categories, catalytic activity (GO:0003824) was the major category with 130 genes enriched. Intracellular (GO:0005622) and response to stimulus (GO:0050896) was the major category of CC and BP, respectively. Among the BP categories, most *FaGST* genes were enriched in the response to plant hormones, Notably, 41 *FaGST* genes were enriched in secondary metabolic process (GO:0019748), indicating they might be involved in the secondary metabolic process.

**Figure 2:**
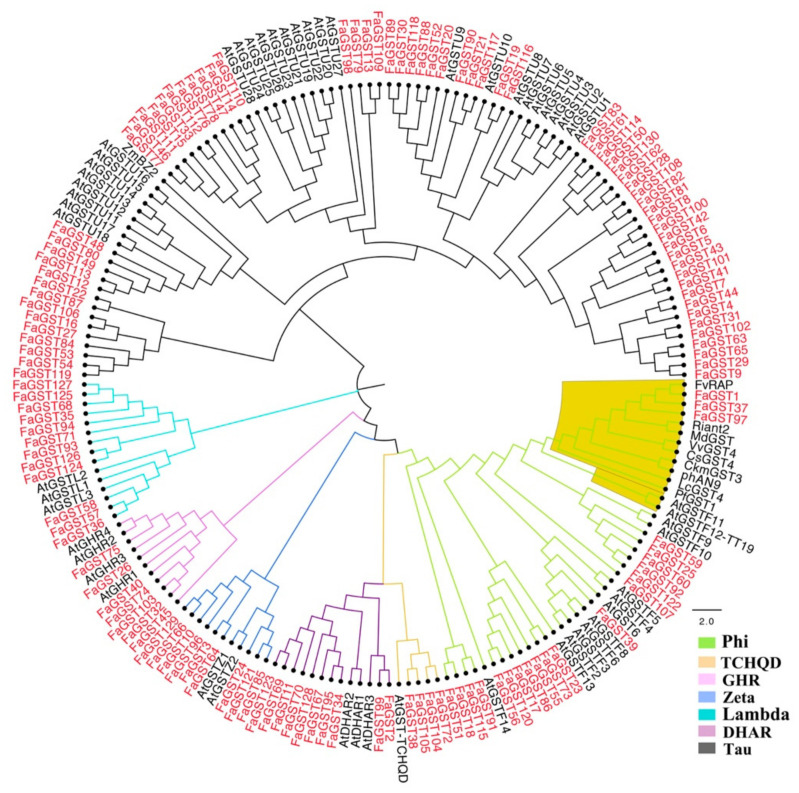
Phylogenetic tree constructed with 130 FaGST protein sequences with the GST from Arabidopsis. The tree was constructed using MEGA X with neighbor-join method. The red and black nodes indicated *GST* genes from strawberry and Arabidopsis. Different colored branches indicate different subclasses of *GST* genes, the highlighted branch indicates the anthocyanin-related *GST* genes as previously proved.

To classify and identify the anthocyanin-related *FaGST* genes in strawberry, a phylogenetic tree (Figure 2) was constructed between 130 *FaGST* genes, 53 *AtGST* genes from *Arabidopsis,* and another nine published anthocyanin-related *GST* from other species based on the protein alignment. As results, these GST proteins were classed into seven classes including Phi, Tau, DHAR, TCHQD, GHR, lambda, and Zeta. The largest class of *FaGST* is Tau followed by Phi. All the previously suggested anthocyanin-related *GST* genes were closely clustered into Phi class, except *BZ2* from maize. Notably, three *FaGST* genes named *FaGST1*, *FaGST37*, and *FaGST98* showed a close relationship with the previous suggested anthocyanin-related *GST* genes, indicating that these three *FaGST* genes might be anthocyanin-related.

### 2.3. Gene Structure and Conserved Motifs Analysis

**Figure 3:**
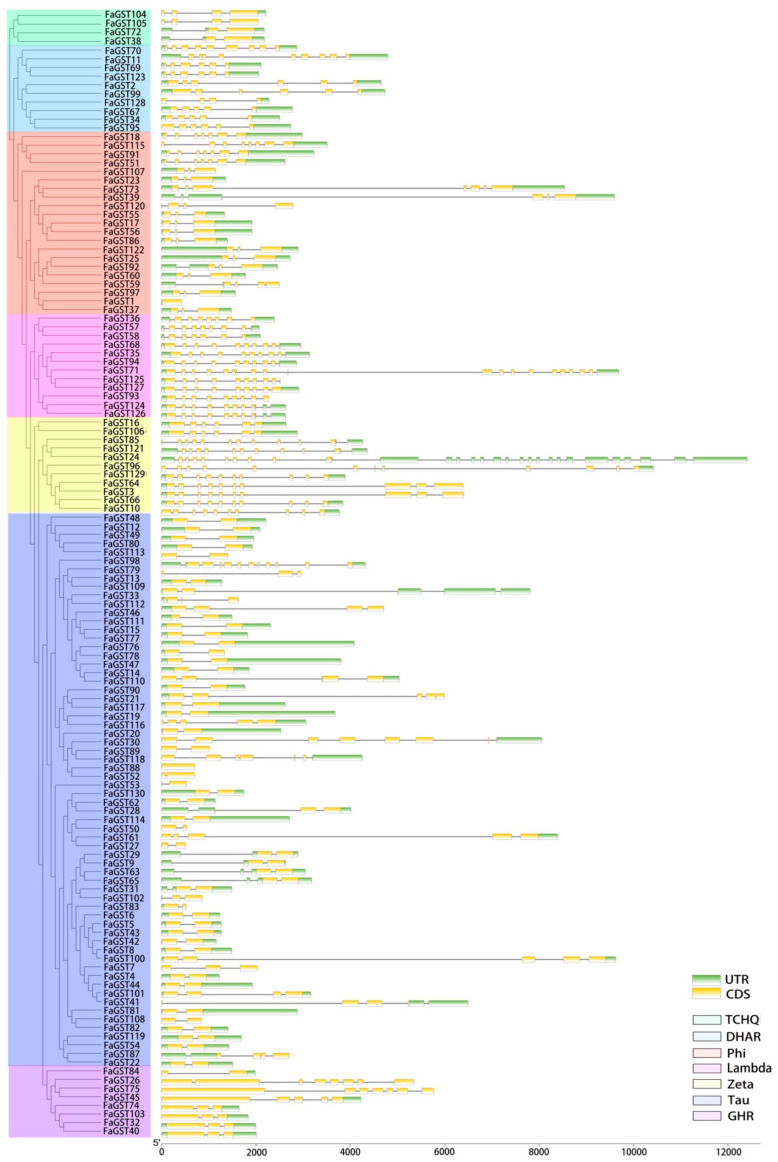
Exon–intron structures of *FaGST* genes. The cladogram was constructed by MEGA X software using the full-length of FaGST proteins. The exons and introns were represented by yellow blocks and black lines, respectively, the green blocks indicated the 5′ and 3′ UTR of the *FaGST* genes. Different colors indicated the different GST classes.

To further explore the structural features of strawberry *GST* genes, the gene structure was analyzed. As results (Figure 3), the Lambda and Zeta class contained the most but shortest exons comparing with other classes. Among the plant-specific Phi class, only *FaGST1* had no intron, 11 of 21 had two introns and the others had more than two introns. For the largest Tau class, most genes had two exons and one intron, *FaGST88* had no intron.

**Figure 4:**
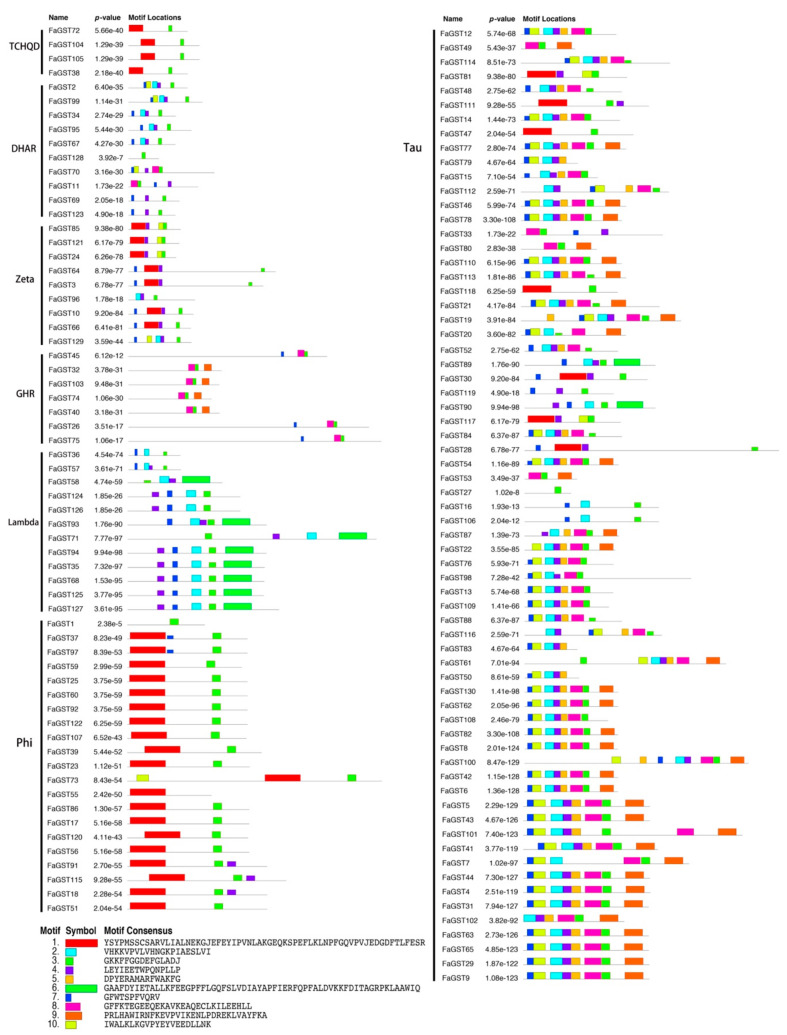
Distribution of the conserved and potential motifs of FaGST proteins. Each motif is represented by colored rectangular box. The core sequence and number of each motif is shown in the black box.

To get more understanding of the functional divergences of the FaGST proteins, a total of 10 motifs in the deduced *FaGST* amino acids were also searched on MEME website. The results (Figure 4) showed that each subclass of *GST* genes comprise the conserved motifs. For instance, almost all the *GST* genes in the Tau class comprised motifs 2, 5, 7, and 9, while motifs 3 and 8 were conserved in the GHR class. Specifically, in the anthocyanin-related class (Phi), motifs 1 and 3 were conserved. The putative motifs were annotated based on NCBI Conserved Domain Database (CDD), Pfam and PROSITE databases. Motifs 1 and 2 were annotated as GST_N domain, motifs 3 and 6 were annotated as GST_C domain.

### 2.4. Expression Analysis of FaGST Genes in Different Developmental Stages

**Figure 5:**
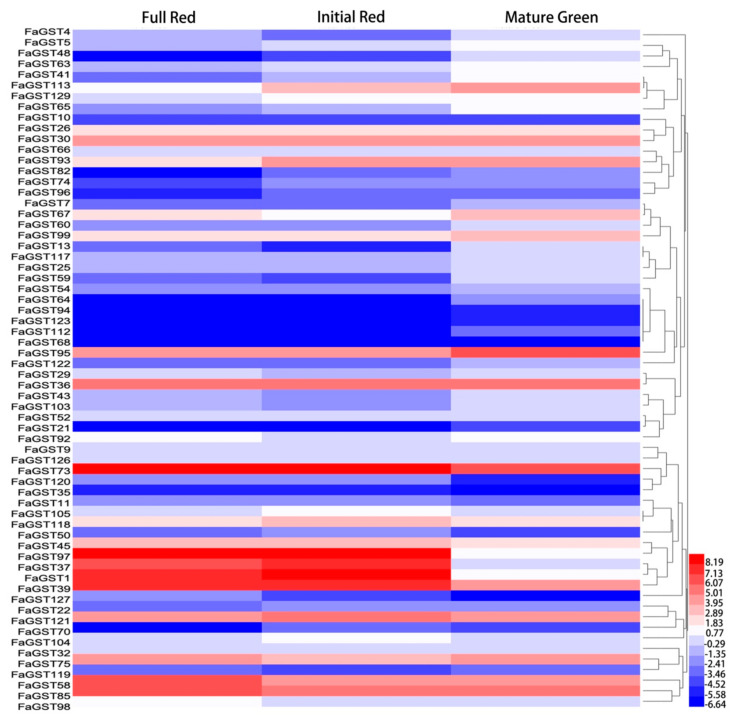
Expression profiles of *FaGST* genes at different developmental stages. RNA-seq based expression profiles corresponding to 87 *FaGST* genes were retrieved from the published RNAseq data (Accession: SRX6381726). The heatmap represented the log2 FPKM value of *FaGST* genes.

To further confirm the involvement of *FaGST* genes in anthocyanin transportation, their expression was analyzed at different developmental stages using transcriptome data. Among the total of 130 *FaGST* genes, expression data of 87 *FaGST* genes were available and retrieved for analysis. As the results (Figure 5), most of the genes were found to be low expressive, and the expression of *FaGST97* at IR was found to be the maximum, followed by *FaGST1* and *FaGST73*. All these genes could be divided into four groups based on their expression patterns during the developmental stages: (a) low expression level at the initial red (IR) stage while comparative higher expression at full red (FR) and mature green (MG), such as *FaGST4*, *FaGST13,* and *FaGST119* etc. (b) Expression level gradually decreased from MG to FR, such as *FaGST5*, *FaGST48*, *FaGST63*, *FaGST41*, *FaGST113*, *FaGST129*, and *FaGST65* etc. (c) Relatively higher expression at FR, lower expression level at IR, and MG, such as *FaGST58*, *FaGST85,* and *FaGST98* etc. (d) Notably, the three genes (*FaGST1*, *FaGST39,* and *FaGST97*) which clustered to the anthocyanin-related clade had very low expression level at MG, while their expression significantly largely increased at IR and maintained a comparative high level at FR stage. In addition, there were another two genes (*FaGST37* and *FaGST73*) belonging to the Phi class that had a similar expression pattern with these three genes, both of which had an increasing expression at IR stage, and a high expression level at FR stage. These results indicated that *FaGST1*, *FaGST39* and *FaGST97*, *FaGST37* and *FaGST73* were putative anthocyanin-related.

**Figure 6:**
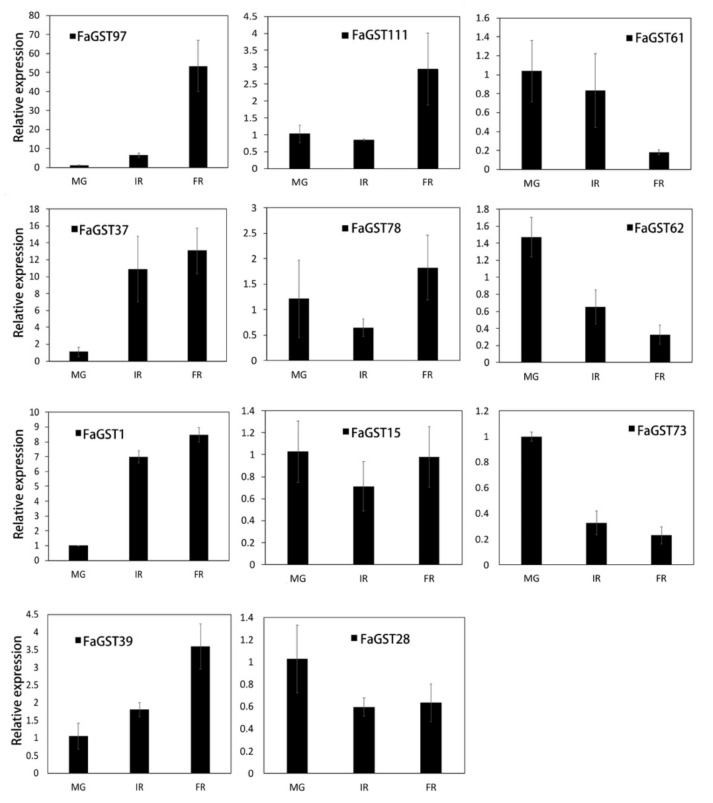
The qPCR-based expression levels of selected *FaGST* genes at different developmental stages. The horizontal axis indicated different developmental stages, MG, mature green; IR, initial red; FR, full red. The bars represented the relative expression of each gene, error bars indicated the standard deviation of three biological replicates. The expression of each gene was normalized against the value at MG stage.

To verify the gene expression data retrieved by RNAseq analysis, the five *FaGST* putative anthocyanin-related genes (*FaGST1*, *FaGST37*, *FaGST39*, *FaGST97*, and *FaGST73*) and another six *FaGST* genes whose expression levels were not available in the RNAseq data were randomly selected for qPCR analysis. The results are shown in Figure 6. It was showed that despite the quantitative differences in expression levels, the expression patterns of the five putative anthocyanin-related *FaGST* genes detected by qPCR experiments were consistent with the expression patterns investigated by RNAseq. In addition, we found another gene named *FaGST111* also had a largely increasing in the FR stage. All the other five *FaGST* genes were detected with constitutive low expression during the whole fruit ripening process. Among them, *FaGST6*, *FaGST28*, and *FaGST62* comprised a gradually decreasing in expression, while *FaGST78* and *FaGST15* showed no big change in expression in different developmental stages according to the qPCR results.

### 2.5. Anthocyanins Accumulation and Expression Analysis of Anthocyanin Biosynthetic Structural Genes

**Figure 7:**
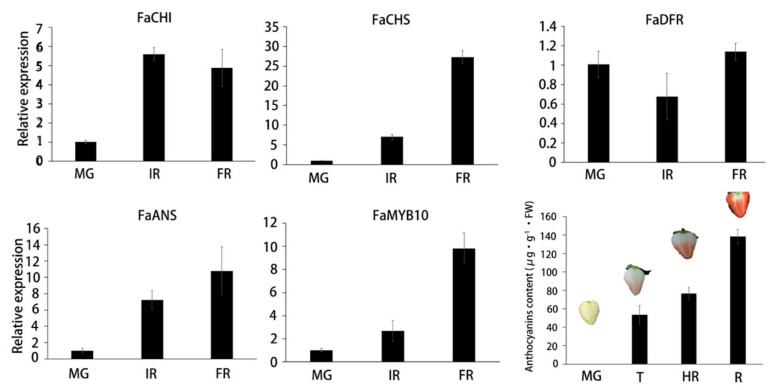
The qPCR-based expression levels of anthocyanin biosynthetic genes and anthocyanin content during fruit ripening. The expression of genes was normalized against the value at MG stage. Horizontal axis indicates the different developmental stages, vertical axis indicates the relative expression. MG, mature green; IR, initial red; HR, half red; FR, full red. Error bars indicate the standard deviation of three biological replicates.

To estimate the relationship between the *FaGST* gene expression and anthocyanin accumulation, the expression of some anthocyanin biosynthesis structural genes and anthocyanin content was detected during the fruit ripening process. The pelargonidin 3-glucoside and cyanidin 3-glucoside have been recognized as two major anthocyanins in strawberry [31]. The content of pelargonidin 3-glucoside was suggested much higher than cyanidin 3-glucoside, it accounts for over 70% of total anthocyanins [32,33]. Here we only detected the pelargonidin 3-glucoside as presentative of total anthocyanins in strawberry. As results (Figure 7), anthocyanins started to accumulate at T stage, and increased gradually during the fruit ripening. Consistent with anthocyanins content, the expression of selected structural genes including *FaCHI*, *FaCHS*, and *FaAN*S increased during the fruit ripening process. Similarly, the expression of *FaMYB10* was also detected as increasing during fruit ripening.

### 2.6. Correlation Analysis of Anthocyanin Biosynthetic Structural Genes and FaGST Genes

**Figure 8:**
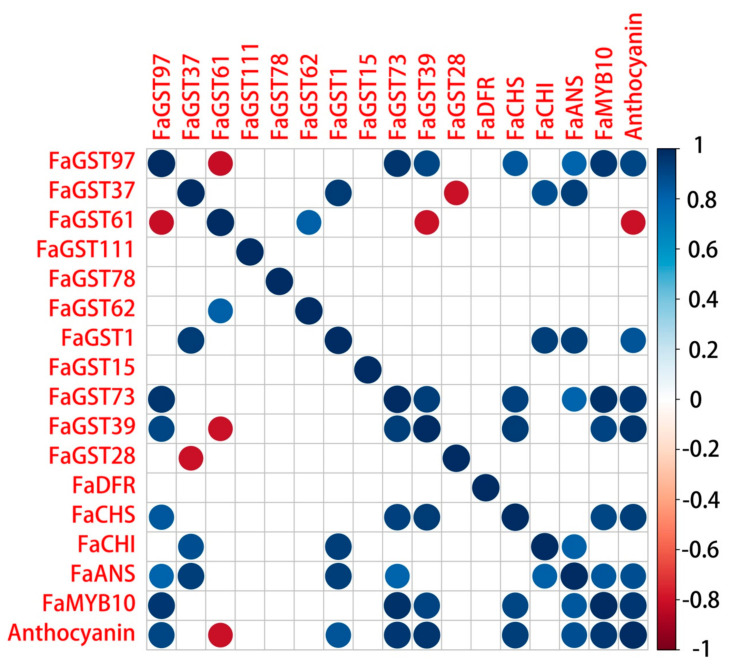
The correlation analysis of selected putative anthocyanin-related *FaGST* genes, anthocyanin biosynthetic/regulatory genes, and anthocyanin content. Dots indicate the significant correlation (Pearson test, *p*-value < 0.05), blank indicates the insignificant correlation. Color from red to blue indicates the correlation coefficient ranging from −1 to 1.

Furthermore, the Pearson correlation between the selected *FaGST* genes and anthocyanin content as well as anthocyanin-related structural genes was analyzed by R software. It was showed (Figure 8) that besides the anthocyanin-related structural genes, the expression of *FaGST97*, *FaGST1*, *FaGST73*, and *FaGST39* was significantly (*p* value < 0.01) strongly positively correlated with anthocyanin content. Their expression was also correlated with the expression of anthocyanin biosynthesis genes during fruit ripening. While, the expression of *FaGST73* has a strong correlation only with the anthocyanin biosynthesis genes *FaCHI* and *FaANS*, but not with anthocyanin content. Unexpectedly, there was a gene named *FaGST61* expressed that was negatively correlated with anthocyanin content. The correlation coefficient value is shown in supplementary Table S2.

### 2.7. Identification of Cis-Regulatory Elements in the Promoters of Putative Anthocyanin-Related FaGST Genes

**Figure 9:**
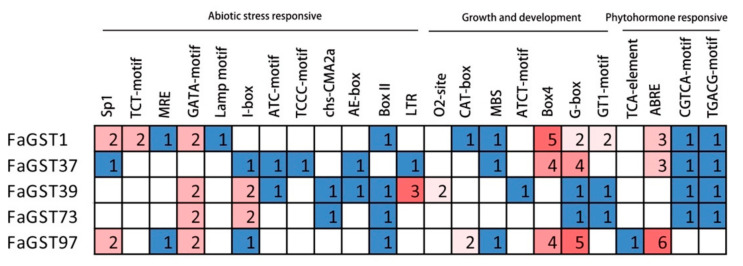
*Cis*-regulatory elements found in the putative anthocyanin-related *FaGST* genes. All the elements were classified into three groups involved in abiotic stress, growth, and development, and phytohormone responsiveness based on the functional annotation.

To investigate the potential regulatory mechanism of *FaGST* genes, the putative promoter regions (2 Kb upstream of the ATG codon) of FaGST genes were submitted to the PlantCARE database. As results (Figure 9, supplementary Table S3), a large number of *cis*-regulatory elements involved in light responsive were identified in the five *FaGST* genes. For example, a total of 17, 13, 10, 6, and 16 light responsive elements (such as Sp1, G-box, GATA-box etc.) were found in the promoter of *FaSGT1, FaGST37, FaGST39, FaGST73,* and *FaGST97,* respectively. Additionally, various regulatory elements involved in hormones responsive were identified. For instance, there were 3, 3, and 6 ABRE elements involved in the abscisic acid responsiveness in the promoter of *FaGST1, FaGST37,* and *FaGST97* genes. Additionally, many *cis*-regulatory elements involved in abiotic stress responsiveness were also identified in the promoter of *FaGST* genes, including *cis*-regulatory element involved in low-temperature responsiveness and MYB binding site involved in drought-inducibility.

## 3. Discussion

### 3.1. Identification and Comprehensive Analysis of the Entire FaGST Genes

GST are encoded by a large superfamily in plants, while the amount of family members vary with species. Previously, 53, 61, and 49 *GST* genes were identified in *Arabidopsis* [34], *Citrus* [35], and melon [36], respectively, while only 32 *GST* genes were identified from pumpkin [37]. In the present study, 130 *FaGST* genes were found in the cultivated strawberry genome, indicating the *GST* family was expanded in strawberry. The expansion of gene families mainly occurred due to various types of gene duplication events such as segmental duplication, tandem duplication, transposition, and whole genome duplication [38]. Gene duplication is an important evolutionary mechanism providing a source of genetic material for the specialization [39,40]. Plant genome has an abundance of duplicate genes, the whole-genome duplication (WGD) also called polyploidization is an extreme mechanism of gene duplication that leads to an increase in both genome size and the entire gene set [41]. In this study, we found that the expansion of *FaGST* family was mainly driven by WGD/segmental duplication event, since 121 out of 130 *FaGST* were involved in WGD/segmental duplication. Our results also showed that the Ka/Ks of almost all the *FaGST* genes were below 1, indicating they were under stable selection. Subcellular localization studies provide potential information about the physiological context of protein function and significant role in the functional divergence of gene families [42]. Predictive subcellular localization analysis of strawberry GST proteins suggested that most of the FaGST proteins were cytoplasmic, few FaGST proteins were predicted to be localized in chloroplast, mitochondria, and nucleus. This is consistent with many other previous studies [38,43], suggesting the conservation of GST subcellular location and functions.

The presence of introns provides evolutionary protein diversity by increasing exon shuffling and alternative splicing [44]. It has been reported that a single intronic nucleotide mutation could provide changes in gene splicing patterns [45]. Our gene structure analysis indicated that each class of *FaGST* genes contained different numbers of introns, suggesting the divergence of alternative splicing of each class. Normally, the *GST* genes belonging to Tau and Phi class contained one-intron/two-exon, two-intron/three-exon structure in higher plants, respectively, while there was also some exceptions [43,46]. Similarly, here we found the most *FaGST* genes belonging to Tau class showed one-intron/two-exon structure pattern, while *FaGST101, FaGST61,* and *FaGST46* presented four exons. In the Phi class, except *FaGST18, FaGST115, FaGST51, FaGST91*, *FaGST73,* and *FaGST39* with more than three exons, the others were in two-intron/three-exon structure pattern. In addition, the catalytic function of *GST* family was shown to be controlled mainly by the residue in the N-terminal domain [47]. In the present study, the conserved motif analysis showed there were 20 *FaGST* genes that do not contain GST_N motif (motif 1 and 2), suggesting most *FaGST* genes were involved in catalytic function, which was validated in the subsequent gene ontology analysis. Interestingly, all the *FaGST* genes belonging to the GHR class contained no N-terminal GST domain, indicating they might have other functions than catalytic function.

### 3.2. Gene Ontology and Classification Revealed the Putative Anthocyanin-Related FaGST Genes

The *GST* genes are involved in multiple functions such as plant growth, stress responses, and detoxification of xenobiotics [48,49]. To investigate the potential functions of *FaGST* genes, gene ontology analysis was carried out in the present study. Consistent with the previous research, our results suggested that catalytic activity was the major MF category, indicating most *FaGST* genes were involved in various catalytic functions. Notably, secondary metabolic process was enriched in the BP, that gave us a clue that *FaGST* might participate in the secondary metabolic biological process. Recently, *GST* genes were also proven to be related to anthocyanin transportation [23], various anthocyanin-related *GST* genes have been clarified to date. They all belong to Phi subclass, except *ZmBz2*, a anthocyanin-related *GST* gene from maize, which was classified into Tau class [50,51]. Similar to other plant species, our phylogenetic analysis showed that 130 *FaGST* genes could be classified into seven subclasses in strawberry. In case of each class, Tau was the most numerous class with 66 members in strawberry, followed by Phi class. All the previous suggested anthocyanin-related *GST* genes were clustered into the same clade belonging to the Phi subclass, indicating the sequence conservation linked to specific functional requirements. Based on this theory, we noticed from our results that there were three *FaGST* genes included in this clade, making it possible that these genes were involved in vacular anthocyanin accumulation. We therefore included these three *FaGST* genes as putative anthocyanin-related *FaGST* genes in strawberry.

### 3.3. Expression and Correlation Analysis Confirmed the Involvement of FaGST in Strawberry Anthocyanins Accumulation

Anthocyanins belonging to the flavonoid class of compounds are synthesized in cytosol and then transferred to vacuole to exhibit the visible color. Several studies have already confirmed the involvement of *GST* genes in the transportation of anthocyanins. Moreover, it is known that anthocyanin-related *GST* genes and anthocyanin biosynthetic genes are coordinately expressed, consistent with anthocyanin accumulation. For instance, transcriptional profiling of *VvGST4* in *Vitis vinifera* was similar to that of the anthocyanin pathway genes, *CHS*, *DFR*, and *UFGT*, indicative of coregulation [52]. In cyclamen, the expression pattern of *CkmGST3* was also correlated with other anthocyanin biosynthetic genes such as *CkmF3′5′H* and *CkmDFR2*, consistent with the developmental stage of flavonoid accumulation tissues [26]. In *Dracaena cambodiana*, the expression of *DcGST4*, *DcGSTU5,* and *DcGSTU9* were correlated to anthocyanin accumulation under inducer treatment, as well as the expression levels of eight anthocyanin biosynthetic structural genes and two regulatory genes [53]. In wildwood strawberry, *RAP* encoding a *GST* gene was developmentally regulated in expression during fruit coloration [30]. In this study, the expression of a number of *FaGST* genes were analyzed based on the reported RNAseq data. The results showed us that besides the three *FaGST* genes (*FaGST1*, *FaGST39,* and *FaGST97*) clustered into the anthocyanin-related clade, the expression of *FaGST37* and *FaGST73* largely increased at the IR stage, when the anthocyanin started accumulating (Figure 7), indicating the expression of these five genes might contribute to the strawberry coloring. This was validated by our subsequent correlation analysis, which showed that expression of these five *FaGST* genes was significantly correlated with anthocyanin accumulation during strawberry ripening except *FaGST37*. Furthermore, to validate the RNAseq expression data, these five *FaGST* genes, and randomly selected six other *FaGST* genes whose expression were unavailable in the RNAseq expression profiles for qPCR analysis were used. The results showed that all of the five putative anthocyanin-related *FaGST* genes showed the similar expression pattern with RNAse profiles, and the other six *FaGST* genes comprised continuous low expression levels at different developmental stages, except *FaGST111*, which showed a large increase at FR stage, indicating it might also be involved in strawberry anthocyanin accumulation. However, according to our correlation analysis, the expression of *FaGST111* did not show any correlation neither with anthocyanin accumulation nor anthocyanin biosynthetic genes. In contrast, the expression of five *FaGST* genes (*FaGST97, FaGST37, FaGST1, FaGST73,* and *FaGST39*) were significantly correlated to those of structural genes (such as *FaCHS*, *FaCHI*, and *FaANS*), suggesting they are indeed involved in anthocyanin accumulation. Interestingly, among these five *FaGST* genes, only *FaGST37* showed no significant correlation with anthocyanin accumulation, indicating it might also comprise other functions, which needs further research.

## 4. Materials and Methods

### 4.1. Plant Materials

Strawberry cultivar ‘Benihoppe’ were grown in a greenhouse located in Shuangliu, Sichuan province, China. The growth condition was controlled at 22 ± 2 °C, relative humidity 70–90%, 16/8-h light/dark regime. Fruits at three developmental stages were collected from at least 10–15 individual plants. The three developmental stages were defined as mature green (MG, ≈18 days post-anthesis, DPA), turning red (TR, ≈24 DPA), half red (HR, ≈28 DPA), and all red (AR, ≈31 DPA). A group of samples from each 3–5 individual plants were mixed as one biological replicate. All samples were grounded into powder in liquid nitrogen and stored at −80 °C until further use.

### 4.2. Identification and Comprehensive Analysis of FaGST Genes

The protein sequence of *Arabidopsis* GST (AT5G17220) downloaded from TAIR (https://www.arabidopsis.org/) was used as query to search against the genome database for strawberry [54] (GDR, https://www.rosaceae.org). The sequence with high score in the results was retrieved as potential GST paralogous in cultivated strawberry (FaGST). The number of amino acids, open reading frame (ORF) length, putative protein molecular weight (MW), and isoelectric point (pI) for each sequence were obtained using ExPASy ProtParam tool (http://web.expasy.org/protparam/). The conserved domains in the GST proteins were screened and annotated based on Pfam [55], NCBI CDD (http://www.ncbi.nlm.nih.gov/Structure/cdd/wrpsb.cgi) [56] and ProSITE databases. The exon–intron structure of the GSTs was analyzed using Gene Structure Display Server v.2.0 (http://gsds.cbi.pku.edu.cn/index.php) based on the alignment of their CDSs with corresponding genomic DNA sequences. To identify the conserved motifs of GST proteins, the MEME online program (http://meme-suite.org/tools/meme) was used with the following parameters: any number of repetition, maximum number of motifs was set as 10, and optimum motif length was set to 6–100 residues. The upstream 2 Kb sequences of each *FaGST* were retrieved from the corresponding genome as the putative promoter regions. The distribution of *cis*-element in the promoter regions were identified using PlantCARE online software (http://bioinformatics.psb.ugent.be/webtools/plantcare/html/). Gene ontology analysis was conducted by TBtools software.

### 4.3. Phylogenetic and Evolutionary Analysis of FaGST Genes in Strawberry

Phylogenetic tree of GST proteins was constructed using Clustal X v.2.0 and MEGA X software [57] with the neighbor-joining (NJ) method [58], 1000 bootstrap test replicates were chosen to evaluate the reliability of interior branches. The *AtGST* sequences used for alignment were downloaded from TAIR. The eight published anthocyanin-related *GST* were downloaded from NCBI with Genbank accession number AB362191 (*PfGST*), KT946768 *(LcGST*), AY971515 (*VvGST4*), AB682678 (*CkmGST3*), Y07721 (*PhAN9*), ABA42223 (*CsGST4*), AEN84869 (*MdGST*), and KT312848 (*Raint2*). Additionally *FvRAP* (gene10500) was downloaded from GDR database. The collinear gene pair was determined using MCScanX software (http://chibba.pgml.uga.edu/mcscan2/). Synonymous (Ks) and nonsynonymous (Ka) substitutions per site between duplicated *FaGST* genes pairs were subsequently calculated using KaKs Calculator v.1.2 software [59] based on the multiple alignment by MEGA X.

### 4.4. Expression Analysis of FaGST Genes in Strawberry

The RNAseq-based expression levels of *FaGST* genes in strawberry were retrieved from the online transcriptomic data (SRA accession: SRX6381727). qPCR-based expression analyses were carried out using SYBR Green Premix Ex TaqTM (Takara, Japan) on a CFX96 qPCR system (Bio-Rad, USA) in triplicate of each sample. Fluorescence was monitored at the end of the annealing step each cycle. Melting curve was inserted, ramping from 65 to 95 °C (increment 0.5 °C/5 s) after the final cycle. The relative expression was calculated using the 2^−ΔΔ*C*t^ method [60]. *Fa26S* rRNA (accession: X58118) was used as the reference gene to standardize the raw data. All primers used for qPCR in the present study are listed in Table S4 (supplementary Table S4).

### 4.5. Measurement of Anthocyanins Content

The anthocyanins content was determined using HPLC based on previously described method [33]. A C18 column was used for HPLC experiment, 95% formic acid and methanol was used as mobile phases. Compound separations were achieved by a 20 min linear gradient (95–0%) of formic acid in methanol. A total of 10 μL of each sample was injected for HPLC analysis, the column temperature was kept at 25 °C, the flow rate was 1 mL/min, and chromatograms were recorded at 510 nm. The anthocyanins were quantified by compared to external standards. Experiments were repeated three times with three independent samples referring to the three biological replicates.

### 4.6. Statistical Analyses

All data presented here are represented by mean ± standard error of three independent biological replicates. The statistical significance of the difference was evaluated by Data Processing System (DPS) using Duncan’s multiple range test at *p* < 0.05.

## 5. Conclusions

In the present study, we identified 130 *FaGST* genes in the genome of cultivated strawberry. The subsequent classification and evolutionary analysis of *FaGST* revealed the *FaGST1*, *FaGST97*, and *FaGST37* were closely clustered with the previous anthocyanin-related *GST* genes, indicating they were anthocyanins-related but it could not exclude the involvement of other *GST* genes in anthocyanins accumulation. Furthermore, the RNAseq-based expression analysis indicated the expression patterns of *FaGST1*, *FaGST97*, *FaGST37*, *FaGST39,* and *FaGST73* were similar to the anthocyanin accumulation during strawberry fruit ripening. Finally, the qPCR validation and correlation analysis confirmed that the expression of *FaGST1*, *FaGST97*, *FaGST39,* and *FaGST73* were significantly correlated with those of anthocyanin biosynthetic structural genes and anthocyanin accumulation, indicating they are involved in anthocyanin accumulation in strawberry.

## Figures and Tables

**Table 1:** Ka/Ks of glutathione S-transferase (*GST*) paralog genes from cultivated strawberry.

Gene1	Gene2	Ka	Ks	Ka/Ks	*p*-Value (Fisher)
FaGST25	FaGST93	0.00200317	0.113747	0.0176108	1.06 × 10^−9^
FaGST2	FaGST100	0.00165273	0.048333	0.0341946	1.84 × 10^−5^
FaGST12	FaGST114	0.00375505	0.0832149	0.0451247	3.23 × 10^−7^
FaGST25	FaGST123	0.00804594	0.0584878	0.137566	0.00106652
FaGST12	FaGST81	0.00375505	0.0246355	0.152424	0.0300419
FaGST6	FaGST42	0.0244688	0.143011	0.171097	6.23 × 10^−7^
FaGST46	FaGST78	0.00492559	0.02814	0.175039	0.0651319
FaGST79	FaGST111	0.00787615	0.0439795	0.179087	0.00889461
FaGST34	FaGST96	0.0102566	0.0560355	0.183037	0.00310094
FaGST26	FaGST76	0.0202591	0.109809	0.184494	6.95 × 10^−19^
FaGST13	FaGST47	0.173126	0.886179	0.195362	4.53 × 10^−17^
FaGST13	FaGST79	0.18648	0.93119	0.20026	5.53 × 10^−17^
FaGST32	FaGST40	0.00760364	0.0369972	0.205519	0.00105106
FaGST18	FaGST92	0.0104022	0.0497516	0.209083	0.00447566
FaGST58	FaGST94	0.313243	1.48572	0.210836	9.04 × 10^−19^
FaGST32	FaGST104	0.0164355	0.0760217	0.216195	5.48 × 10^−6^
FaGST33	FaGST113	0.0280173	0.128595	0.217873	0.000335365
FaGST4	FaGST44	0.0471441	0.216188	0.21807	2.45 × 10^−7^
FaGST22	FaGST54	0.30599	1.31495	0.232701	8.57 × 10^−16^
FaGST71	FaGST125	0.0254999	0.0973965	0.261815	0.000205114
FaGST15	FaGST112	0.0116752	0.0444317	0.262766	0.0242682
FaGST29	FaGST9	0.00382776	0.0142017	0.269529	0.340562
FaGST29	FaGST65	0.0333278	0.122567	0.271916	0.000450127
FaGST18	FaGST116	0.0113206	0.0411117	0.275361	0.0294135
FaGST10	FaGST66	0.00308679	0.0103946	0.29696	0.355313
FaGST38	FaGST105	0.0127675	0.0425923	0.29976	0.0330291
FaGST36	FaGST57	0.0244337	0.0796125	0.306908	0.00426484
FaGST16	FaGST107	0.0136969	0.0414432	0.330497	0.0194532
FaGST20	FaGST118	0.332674	0.989292	0.336274	1.33 × 10^−10^
FaGST15	FaGST46	0.00987768	0.0282057	0.350201	0.234251
FaGST18	FaGST51	0.0145916	0.0410609	0.355365	0.0477493
FaGST34	FaGST67	0.0425944	0.117162	0.363551	0.00353968
FaGST26	FaGST45	0.0827313	0.226323	0.365546	4.19 × 10^−14^
FaGST1	FaGST37	0.0221609	0.0555156	0.399184	0.124764
FaGST23	FaGST74	0.0150372	0.0375368	0.400598	0.0659631
FaGST36	FaGST58	0.0337835	0.0776749	0.434935	0.0314613
FaGST38	FaGST73	0.00794979	0.0179958	0.441758	0.281114
FaGST24	FaGST122	0.884215	1.82613	0.484203	0.000255179
FaGST24	FaGST86	0.919184	1.72577	0.532623	0.000805302
FaGST11	FaGST124	0.0431261	0.0770174	0.559953	0.117755
FaGST34	FaGST129	0.171667	0.292162	0.587576	0.0453954
FaGST10	FaGST130	0.0303226	0.0492918	0.615164	0.251029
FaGST13	FaGST110	0.0505542	0.0807071	0.626392	0.190343
FaGST17	FaGST121	0.0854666	0.134315	0.636314	0.127269
FaGST11	FaGST70	0.0894935	0.139369	0.642133	0.065153
FaGST6	FaGST101	0.0314943	0.0481056	0.65469	0.319248
FaGST31	FaGST103	0.0558196	0.084181	0.66309	0.366314
FaGST19	FaGST91	0.286378	0.421856	0.678852	0.0104161
FaGST35	FaGST95	0.0128558	0.0185847	0.691739	0.499311
FaGST22	FaGST88	0.0768175	0.106394	0.72201	0.279675
FaGST30	FaGST89	0.177649	0.243199	0.730469	0.117698
FaGST35	FaGST126	0.0173247	0.0233682	0.74138	0.562354
FaGST10	FaGST97	0.083706	0.111366	0.751627	0.358856
FaGST12	FaGST48	0.0844299	0.111337	0.758326	0.31708
FaGST52	FaGST119	0.249063	0.327341	0.760868	0.178488
FaGST30	FaGST52	0.220105	0.288024	0.764189	0.168512
FaGST35	FaGST71	0.0215799	0.0279284	0.772687	0.603323
FaGST32	FaGST75	0.167423	0.216594	0.772981	0.131165
FaGST17	FaGST87	0.0160792	0.0205336	0.783069	0.683377
FaGST35	FaGST128	0.0208453	0.0255813	0.814864	0.591106
FaGST50	FaGST115	0.0302207	0.035657	0.84754	0.701964
FaGST1	FaGST98	0.0286463	0.0326903	0.876291	0.710666
FaGST27	FaGST61	0.17827	0.198328	0.898862	0.724287
FaGST35	FaGST68	0.017273	0.0138736	1.24503	0.989446

## Supplementary Materials

The following are available online at https://www.mdpi.com/1422-0067/21/22/8708/s1. Table S1. The basic information of *FaGST* genes in cultivated strawberry. Table S2. The coefficient value and *p*-value of correlation analysis. Table S3. Cis-regulatory elements identified in the putative anthocyanins-related *FaGST* genes. Table S4. Lists of all primers used for qPCR analysis in this study. Figure S1. Chrome distribution of *FaGST* genes in the cultivated strawberry genome.
