# Supplementary figures and images for: Identification of Anthocyanins-Related Glutathione S-Transferase (GST) Genes in the Genome of Cultivated Strawberry (Fragaria × ananassa)
Source: Int J Mol Sci. 2020 Nov 18;21(22):8708. doi: 10.3390/ijms21228708 (PMC7698900; doi:10.3390/ijms21228708)

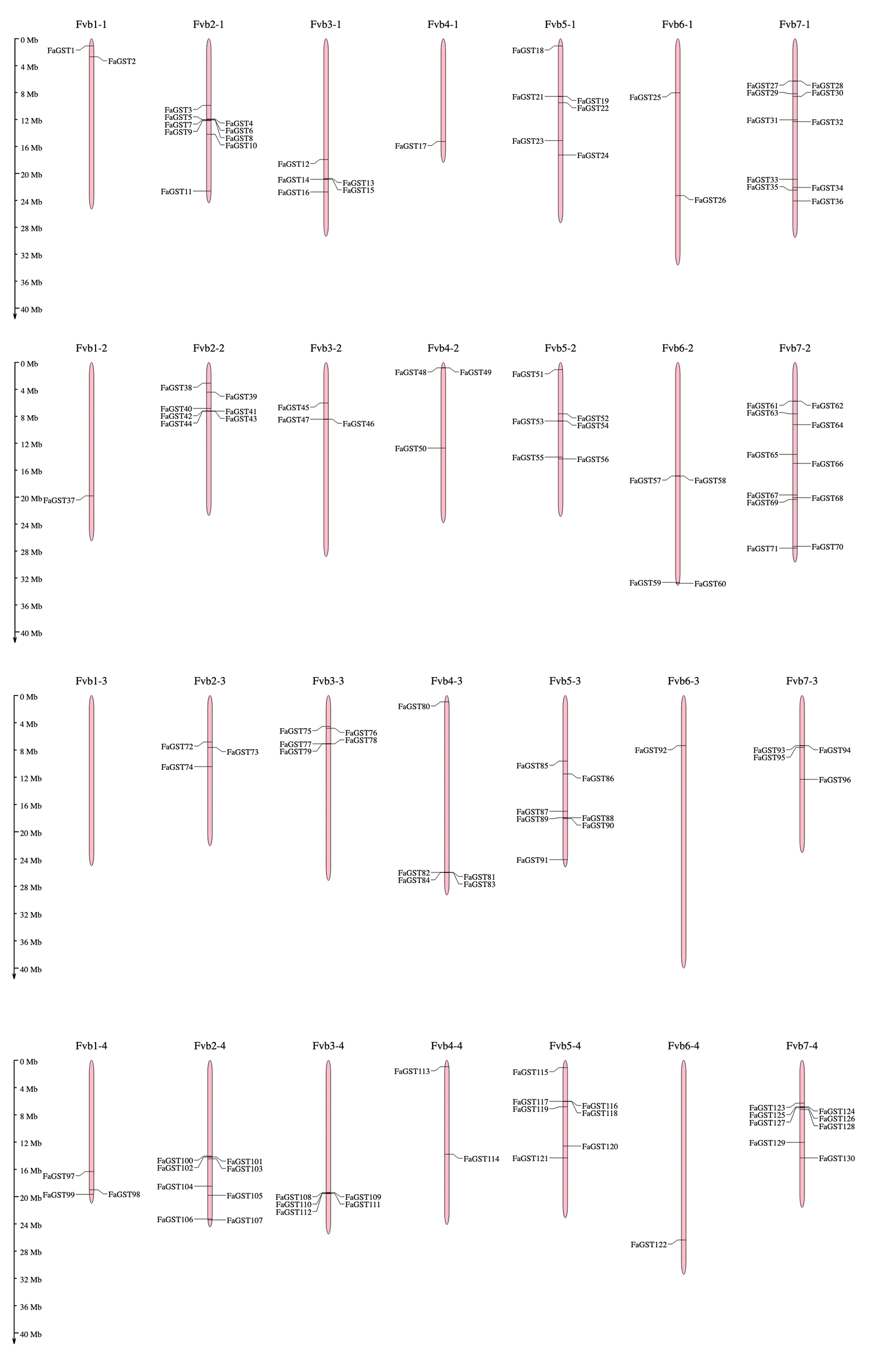

Supplement: Supplementary file 1 [file ijms-21-08708-s001.zip › Figure S1.jpg]
